# Implementation of a six-month produce prescription program for a rural Medicaid population increased vegetable consumption and reduced food insecurity in Washington state

**DOI:** 10.1016/j.pmedr.2026.103618

**Published:** 2026-09-04

**Authors:** Alyssa Castilla, Guadalupe Silva, Tafere Belay, Deborah Gauck, Nicole Stendell-Hollis

**Affiliations:** a Central Washington University, 400 E. University Way, Ellensburg, WA, USA; b Deborah Gauck Consulting, 413 Adams Creek Place, Hood River, OR, USA

## Abstract

**Objective:**

Medicaid recipients in rural populations experience higher rates of food insecurity. Produce prescription (PRx) programs subsidize the cost of fruits and vegetables (F/V) and may improve health outcomes. This study was designed to test whether a PRx program using a community supported agriculture (CSA) model would improve health outcomes and reduce healthcare utilization among rural Medicaid patients with/at risk of chronic illnesses.

**Methods:**

A federally qualified health center referred patients to a food bank, which delivered CSA boxes with produce to them for six months. A total of 136 participants completed the baseline survey, and 73 participants completed the post-survey. Main outcomes include F/V intake, food security status, and healthcare utilization. Descriptive statistics were used to summarize demographic characteristics. Chi-squared tests and paired *t*-tests were conducted to assess associations and mean differences.

**Results:**

Daily fruit intake and health status did not significantly change. Significant outcomes include increased vegetable consumption (*p* = 0.04); and decreased food insecurity status (84% to 66%; *p* = 0.03).

**Conclusions:**

These findings suggest that PRx programs can be effective nutrition interventions for rural Medicaid populations. This insight can help inform health providers and policymakers about strategies to address chronic disease management and treatment.

## Highlights

• A produce prescription program in a rural community receiving Medicaid.
• Daily vegetable consumption significantly increased.
• Food insecurity significantly decreased.

## 1 Introduction

The use of food is medicine (FIM) interventions has emerged as a promising strategy to prevent, manage, and treat chronic diseases (Bryce et al., 2017; Downer et al., 2020; Swartz, 2018). Often targeting underserved populations, FIM interventions provide financial support for nourishing foods or direct provision of healthy foods including medically tailored meals, medically tailored groceries, and produce prescription (PRx) programs (Downer et al., 2020; Hager et al., 2024). PRx programs provide fruits and vegetables (FV) through various approaches, including voucher systems, garden-based initiatives, cash-back rebates, subsidized food boxes, and collaborative food pantry-clinical programs (Hager and Mozaffarian, 2020; Veldheer et al., 2020). Despite the recognized benefits of promoting greater FV consumption (Bryce et al., 2017; Sastre et al., 2023; Trapl, 2018), there is a notable gap in research demonstrating how PRx programs improve health outcomes in rural communities (Cafer et al., 2023).

Individuals in rural communities as well as those receiving Medicaid are at a higher risk of food insecurity due to transportation barriers as well as cost challenges to accessing healthier food options (Chapel et al., 2017). These populations continue to see an increase in diet-related health conditions, such as type 2 diabetes, hypertension, and overweight or obesity, as well as increased healthcare utilization costs associated with managing their conditions (Berkowitz et al., 2018; McQueen et al., 2022). Given that the Medicaid population was over 70 million in June 2025 (Medicaid.gov, 2025), PRx programs that focus on this population could significantly impact chronic illness rates.

Community supported agriculture (CSA) is one of several models within the broader category of PRx programs that enables individuals to obtain a small share of produce from local farms and receive fresh FV throughout the season (Izumi et al., 2020). Studies of this model have shown promising results such as better blood glucose control within 13 weeks among patients with a history of uncontrolled diabetes (Bryce et al., 2017), increased consumption of FV and improved glycemic control among uninsured patients with diabetes (Sastre et al., 2023), and increased FV consumption among hypertensive adults (Trapl, 2018). Subsidized CSA programs aid in produce share costs, particularly benefiting participants with lower incomes and higher levels of food insecurity (Lou et al., 2024; Quandt et al., 2013).

This study aims to examine the outcomes and effectiveness of a six-month PRx program utilizing CSA shares for a rural Medicaid population in Kittitas County, Washington. Compared to residents in Washington state, a higher percentage of residents in Kittitas County have food insecurity (12.8% compared to 10.9%), have limited access to healthy foods, are a food desert population, have poor or fair health, have poor physical health days, and have frequent physical distress (Kittitas, Washington, 2025; Kittitas County Public Health Department, 2013; Kittitas County Chronic Disease and Risk Factor Profile, 2025). The project aims were to increase FV consumption, food security, and self-reported health status; and decrease missed health clinic appointments of patients with diet-related health conditions.

## 2 Methods

### 2.1 Study design and population

The six-month PRx program, a pre-post intervention study, adapted the evidence-based CSA Partnerships for Health model, a subsidized CSA program (Izumi et al., 2020). The USDA Gus Schumacher Nutrition Incentive Program (GusNIP) funded the program. A federally qualified health center, food bank, food hub, public health department, and university met monthly to collaborate on the project.

The food hub delivered CSA boxes to the food bank, which distributed them to participants via pick-up or home delivery. CSA boxes included a variety of FV from local farms and had an approximate value of $30. The food bank also provided two tastings of FV, biweekly newsletters about FV, and written and pictorial instructions for preparing vegetables and produce storage and care. These supplementary materials were intended to increase comprehension of the importance of FV consumption and skill in preparing FV.

Participants received additional educational resources, including a cooking demonstration video and two hands-on cooking demonstrations (i.e. Mediterranean diet, flexitarian diet) led by a chef and a registered dietitian nutritionist. Participants practiced cooking skills by preparing healthy meals aligned with these themes and enjoyed meals together. Additionally, the federally qualified health center provided motivational support and addressed patient participation barriers.

Participants were recruited by the federally qualified health center in rural Washington state. The intervention lasted for a six-month duration and participants were enrolled on a rolling basis over the course of one year. Patients were eligible if they were receiving Medicaid and met one or more of the following criteria: (1) had, or were at risk for, a diet-related chronic illness such as diabetes, hypertension, overweight/obesity, gestational diabetes, high cholesterol, and/or severe osteoarthritis; (2) were currently undergoing treatment for opioid use; (3) were pregnant; and/or (4) were up to 12 months postpartum. Family members of eligible participants were also allowed to participate in the study; however, data was only collected for the eligible participant. The federally qualified health center analyzed practice management and electronic health record data to target and verify participants according to eligibility criteria.

### 2.2 Measures

A single, validated, standardized survey was administered pre- and post-program (within one month of study conclusion) by trained study staff and included: 1) the Nutrition Incentive Program Training, Technical Assistance, Evaluation, and Information Center (NTAE) Participant-Level Survey (Nutrition Incentive Hub, 2025); 2) the USDA 6-item Short Form Household Food Security Survey Module (HFSSM); 3) the Dietary Screener Questionnaire (DSQ); and 4) two questions from the National Cancer Institute Food Attitudes and Behavior Survey to assess general health and program satisfaction (Food Attitudes and Behaviors Survey, 2009). Participants had the option to complete surveys on paper or online upon delivery of first food box after eligibility was established; and via paper surveys mailed back, completed online, or over the phone post-survey. The Participant-Level Survey measured participants' basic sociodemographic information, food security, FV consumption, and perceived health status. Food security was measured using the USDA 6-item Short Form Household Food Security Survey Module (HFSSM) (Nutrition Incentive Hub, 2025). FV consumption was measured using ten questions from the Dietary Screener Questionnaire (DSQ) developed by the National Cancer Institute. Perceived health status was assessed using a single question: “Would you say in general that your health is poor, fair, good, very good, or excellent?” Post-program, participants rated their program satisfaction on a 5-point Likert scale from “very negative” to “very positive.” In addition, the federally qualified health center collected appointment no-show data from March to August 2024 and September 2024 to February 2025.

### 2.3 Statistical analysis

Descriptive statistics, including frequency distributions, percentages, and means with standard deviations (SD), were used to summarize the socio-economic and demographic characteristics of respondents as well as food security, health status, healthcare utilization, and program satisfaction. The Chi-squared test was employed to examine associations in the frequency of food intake before and after the intervention. A paired *t*-test was conducted to assess mean differences in FV intake pre- and post-intervention. Consumption frequency was originally assessed using categories ranging from “never” to “2 or more per day”. Responses of “don't know” and/or “prefer not to answer” were excluded from the analysis. The remaining frequency responses were converted to a standardized daily consumption frequency using the National Cancer Institute (NCI) methodology. The resulting daily frequency measure was treated as a quantitative variable for consumption between groups using the paired *t*-test. All analyses were performed using SPSS software (version 30; IBM, Armonk, NY), with statistical significance set at *p* < 0.05.

The Human Subjects Review Council at Central Washington University approved the study before commencement and informed consent was obtained from all study participants.

## 3 Results

### 3.1 Participant-level surveys

A total of 136 participants answered the baseline questionnaire, and a total of 73 participants answered the post questionnaire. Missing data responses were excluded from the analysis. As shown in Table 1, most participants at pre and post were female, white, and non-Hispanic. For the baseline survey, the majority of participants were in the 45–64 age group (37.9%), followed by the 35–44 (23.5%) and 65+ (18.2%) age groups. For the post-survey, the majority of participants were in the 45–64 age group (35.2%), followed by the 65+ (23.9%) and 35–44 (21.1%) age groups.

**Table 1:** Demographic and socioeconomic characteristics of adults enrolled in a six-month produce prescription program for a rural Medicaid population in Washington state.

	Pre (*n* = 136)	Post (*n* = 73)
Variables	n (%)	n (%)
Age (years)^a^		
• ≤18	3 (2.3)	1 (1.4)
• 18–24	4 (3.0)	2 (2.8)
• 25–34	20 (15.2)	11 (15.5)
• 35–44	31 (23.5)	15 (21.1)
• 45–64	50 (37.9)	25 (35.2)
• ≥65	24 (18.2)	17 (23.9)

Gender^a^		
• Male	34 (25.2)	17 (23.6)
• Female	98 (72.6)	53 (73.6)
• Prefer not to answer	2 (1.5)	1 (1.4)
• Non-binary/third gender	1 (0.7)	1 (1.4)

Ethnicity^a^		
• Not Hispanic or Latino/a/x	114 (84.4)	62 (86.1)
• Hispanic or Latino/a/x	16 (11.9)	8 (11.1)
• Prefer not to answer	5 (3.7)	2 (2.8)

Race^a^		
• American Indian or Alaska Native	10 (7.4)	4 (5.6)
• Black or African American	4 (3.0)	2 (2.8)
• White	88 (65.2)	47 (65.3)
• Other	0 (0.0)	3 (4.2)
• More than one race	16 (11.9)	10 (13.9)
• Don't know/not sure	3 (2.2)	2 (2.8)
• Prefer not to answer	14 (10.4)	4 (5.6)

Received EBT, food stamps, or SNAP benefits in the past 30 days^b^		
• Yes	101 (74.2)	60 (82.2)
• No	33 (24.3)	13 (17.8)
• Don't know/Prefer not to answer	2 (1.5)	0 (0.0)

Food insecurity status^b^		
• Very low food security	54 (40.0)	22 (30.6)
• Low food security	54 (40.0)	29 (40.3)
• Marginal food security	13 (9.6)	11 (15.3)
• High food security	14 (10.4)	10 (13.9)

Health status^c^		
• Poor	30 (22.2)	12 (16.7)
• Fair	60 (44.4)	29 (40.3)
• Good	27 (20.0)	26 (36.1)
• Very good	12 (8.9)	4 (5.6)
• Excellent	2 (1.5)	0 (0.0)
• Don't know/Prefer not to answer	4 (3.0)	1 (1.4)

Data collected from the NTAE^a^, the HFSSM^b^, and the NCI Food Attitudes and Behavior Survey^c^.

A chi-squared test was used to assess the relationship between food intake before and after the program. A total of 58 matched individuals completed the pre and post assessments. Consumption was grouped by the following responses: I ate [food type] never to 2–3 times last month, 1–4 times per week, or 5–6 times per week to 6 or more times per day. These groupings were chosen to reflect typical consumption patterns based on infrequent, moderate, or daily intake. Overall, participants' average daily intake in cups of FV increased from 2.28 to 2.46 times per week (data not shown). Daily fruit consumption did not significantly increase; however, daily vegetable consumption significantly increased by 0.13% (*p* = 0.04) (data not shown). Changes in salad (*p* < 0.01) and overall vegetable (*p* = 0.04) intake were statistically significant (data not shown). On the other hand, there were no significant changes in fruit juice, fruit, fried potato, non-fried potato, beans, salsa, pizza, or tomato sauce (data not shown).

Data in Table 2 has been converted from categorical frequency intake to estimated daily frequency (times per day). Daily frequencies of less than 0.083 times per day correspond to categorical frequencies of never to 2–3 times last month (less than once per week), whereas daily frequencies of more than 0.143 times per day correspond to categorical frequencies of more than once per week, representing daily frequency values of 0.143 to 6 or more times per day for fruit juice and 0.143 to 2 or more times per day for foods. Significant findings include salad (*p* < 0.01), beans (*p* < 0.05), and vegetables (*p* = 0.02) (Table 2).

**Table 2:** Changes in daily frequency before and after intervention in a six-month produce prescription program for a rural Medicaid population in Washington state (*n* = 58)^a^, ^b^.

	Pre	Post	
Variable	n (%)	n (%)	p value
Fruit juice			0.42
• ≤0.083 times per day (<1 time per week)	32 (60.4)	29 (52.7)	
• ≥0.143 times per day (≥1 time per week)	21 (39.6)	26 (47.3)	

Fruit			0.61
• ≤0.083 times per day (<1 time per week)	10 (18.2)	8 (14.5)	
• ≥0.143 times per day (≥1 time per week)	45 (81.8)	47 (85.5)	

Salad			<0.01
• ≤0.083 times per day (<1 time per week)	35 (62.5)	18 (33.3)	
• ≥0.143 times per day (≥1 time per week)	21 (37.5)	36 (66.7)	

Fried potato			0.88
• ≤0.083 times per day (<1 time per week)	35 (61.4)	33 (60.0)	
• ≥0.143 times per day (≥1 time per week)	22 (38.6)	22 (40.0)	

Non-fried potato			0.09
• ≤0.083 times per day (<1 time per week)	32 (56.1)	22 (40.0)	
• ≥0.143 times per day (≥1 time per week)	25 (43.9)	33 (60.0)	

Beans			0.05
• ≤0.083 times per day (<1 time per week)	34 (59.6)	22 (40.7)	
• ≥0.143 times per day (≥1 time per week)	23 (40.4)	32 (59.3)	

Vegetable			0.02
• ≤0.083 times per day (<1 time per week)	15 (26.3)	5 (8.9)	
• ≥0.143 times per day (≥1 time per week)	42 (73.7)	51 (91.1)	

Salsa			0.47
• ≤0.083 times per day (<1 time per week)	43 (75.4)	36 (69.2)	
• ≥0.143 times per day (≥1 time per week)	14 (24.6)	16 (30.8)	

Pizza			0.41
• ≤0.083 times per day (<1 time per week)	51 (89.5)	48 (84.2)	
• ≥0.143 times per day (≥1 time per week)	6 (10.5)	9 (15.8)	

Tomato sauce			0.85
• ≤0.083 times per day (<1 time per week)	35 (62.5)	34 (60.7)	
• ≥0.143 times per day (≥1 time per week)	21 (37.5)	22 (39.3)	

a Data derived from the National Cancer Institute for converting frequency data to daily frequency (National Cancer Institute, 2024).

b Daily frequency categorization at ≤0.083 and ≥0.143 times per day is unaffected by differences in conversion values for foods and beverages; intermediate values do not occur.

In Table 3, data on self-reported health status and food security status before and after the intervention are shown. At baseline, participants self-reported poor (25.5%), fair (41.8%), and good, very good, and excellent (32.7%) health status (Table 3). Although not statistically significant, self-reported good, very good, and excellent health status increased by 4.8%, fair increased by 1.1%, and poor decreased by 5.9% (Table 3). Food insecurity status among those who completed pre- and post-program surveys significantly decreased by 17.5% (*p* = 0.03) (Table 3).

**Table 3:** Change in frequency of health status and food security pre-and post-intervention of a six-month produce prescription program for a rural Medicaid population in Washington state.

	Pre (*n* = 58⁎)	Post (n = 58)	
Variable	n (%)	n (%)	p value⁎⁎
Health status			0.74
• Good, very good, excellent	18 (32.7)	21 (37.5)	
• Fair	23 (41.8)	24 (42.9)	
• Poor	14(25.5)	11 (19.6)	

Food security			0.03
• Food secure	9 (15.8)	19 (33.3)	
• Food insecure	48 (84.2)	38 (66.7)	

⁎ Complete pre- and post-data on these questions was only obtained from 58 out of the 73 completers.

⁎⁎ Statistical significance set at *p* < 0.05.

A paired *t*-test was conducted to compare fruit and vegetable FV consumption before and after the intervention. The frequency of salad consumption increased significantly from pre- to post-intervention (*p* = 0.02) (Table 4). Significant increases were also observed in non-fried potato intake (*p* < 0.01), bean consumption (*p* = 0.01), and overall vegetable intake (*p* < 0.01). However, no significant changes were found for fruit juice, whole fruit, fried potatoes, salsa, pizza, or tomato sauce (Table 4).

**Table 4:** Mean Differences in Frequencies of Fruits and Vegetables Between Pre-and Post-Intervention in a Produce Prescription Program (*n* = 58).

	Pre (*n* = 58)	Post (n = 58)	
Variables	Mean (SD)	Mean (SD)	p value
Fruit juice^a^	2.5 (2.6)	3.1 (2.7)	0.14
Fruit^b^	4.9 (2.2)	5.1 (1.8)	0.63
Salad	2.8 (2.3)	3.6 (2.1)	0.01
Fried potato	2.4 (1.8)	2.3 (1.5)	0.64
Non-fried potato	2.3 (1.6)	3.2 (1.5)	<0.01
Beans	2.5 (2.0)	3.3 (1.8)	0.01
Vegetables	4.3 (2.3)	5.2 (1.7)	<0.01
Salsa	1.7 (1.8)	1.9 (2.0)	0.60
Pizza	1.3 (1.1)	1.5 (1.2)	0.19
Tomato sauce	2.2 (1.7)	2.4 (1.3)	0.22

a Fruit juice frequencies were coded as follows: 0 = never; 1 = 1 time last month; 2 = 2–3 times last month; 3 = 1 time per week; 4 = 2 times per week; 5 = 3–4 times per week; 6 = 5–6 times per week; 7 = 1 time per day; 8 = 2–3 times per day; 9 = 4–5 times per day; 10 = 6 or more times per day; 11 = don't know/prefer not to answer.

b Food frequencies were coded as follows: 0 = never; 1 = 1 time last month; 2 = 2–3 times last month; 3 = 1 time per week; 4 = 2 times per week; 5 = 3–4 times per week; 6 = 5–6 times per week; 7 = 1 time per day; 8 = 2 or more times per day; 9 = don't know/prefer not to answer.

Overall program satisfaction was positive (29.8%) and very positive (52.6%), with one participant (1.8%) recording a negative experience and another (1.8%) preferring not to answer (data not shown). There were no significant changes in appointment no-shows (data not shown).

## 4 Discussion

This study presents results from a six-month PRx intervention aimed at increasing FV consumption, improving food security and self-reported health status, and reducing appointment no-shows for Medicaid individuals residing in a rural area. Paired *t*-tests demonstrated significant improvements, including increased consumption of salad, non-fried potatoes, beans, and vegetables. Chi-squared analyses of daily frequency patterns reflected comparable statistical significance for salad, beans, and vegetables. Furthermore, daily vegetable consumption significantly increased (*p* < 0.04), however daily fruit consumption did not. This contrasts with findings from previous produce prescription programs (Ridberg et al., 2019; Spees et al., 2016) that found significant increases in both FV intake. More broadly, findings across diverse populations indicate that FIM interventions are effective in promoting healthier eating habits.

Despite a statistically significant increase, these findings are not clinically significant because daily FV intake remained below recommended levels. The primary limitations of this study included reliance on self-reported assessments rather than objective anthropometric or clinical measures (e.g., BMI, blood pressure, blood glucose), limited participation in nutrition education activities, absence of a control group, and incomplete post-intervention questionnaire data (some participants were lost to follow-up and could not be reached to complete the post-survey). Furthermore, the daily consumption measure was derived from abounded frequency scale. Although it was converted to a quantitative daily-frequency measure for analysis, the bounded nature of the measure may limit strict adherence to the assumptions of the *t*-test and should be considered when interpreting the findings.

FV intake and health status were self-reported, which may have affected the accuracy and reliability of the data. Incorporating clinical measurements in future studies would improve data accuracy and provide objective assessments. To improve data quality while addressing program costs and scalability, future PRx studies may consider exploring Electronic Benefit Transfer (EBT) transaction data (Fischer et al., 2022). Tracking purchases through EBT transactions, rather than preassembling a box of FV, allows participants more autonomy in their food choices and offers valuable insight into outcomes related to FV intake and purchasing patterns.

Participants desired detailed newsletters that included photos for easier identification of FV, more recipes, and comprehensive information about all produce included in the box. Many participants faced barriers in accessing the cooking video, as they were unfamiliar with QR codes, lacked smartphones, or had low vision concerns. Furthermore, the program did not ask about allergies, and one patient reported receiving produce they were allergic to. Further studies should explore ways to improve communication about program offerings and address household-specific needs to enhance participation in and overall effectiveness of PRx programs.

Future studies should employ well-designed programs that include control groups to limit bias and improve generalizability. Of the 73 participants who completed the post-intervention assessments, only 58 could be matched to baseline data. This discrepancy is reflective of the lower participation during the post period. Unmatched data was excluded from the pre-post analysis which may have affected the results, as well as participant responses with missing values for specific questions. Future studies could employ a completion incentive to increase response rates. Additionally, future PRx programs should consider providing an electronic benefits transfer (EBT) card where participants can self-select fruits and vegetables perhaps leading to increased consumption, as most by not all studies show they result in increased fruit and vegetable intake (Owoputi et al., 2025).

## 5 Conclusion

Participants received bi-weekly CSA boxes of FV, along with nutrition education, and completed surveys and interviews to report FV consumption, food security, and perceived health status. Findings showed that the PRx intervention helped increase vegetable intake, improve food security status and perceived health status, and was well received by participants. The study targeted a specific rural Medicaid population and was tailored to individuals who had or were at risk for developing a diet-related illness; thus, our findings may not be generalizable to the U.S. population. Furthermore, the long-term effects of the program remain uncertain. It is unknown whether the changes in participant behavior will be maintained after program completion. Investigating post-intervention effects on FV consumption could provide important insights on how to improve health status for individuals living with chronic illnesses.

Other dimensions of PRx interventions remain challenging to evaluate and several studies push for consistency across models, implementing standardized programs (Cafer et al., 2023), and further exploring methods to assess effectiveness (Little et al., 2021).

## Generative AI usage

During the preparation of this work, AC and LS used generative AI tools to assist with the language flow and readability of the work. After using this service, the authors reviewed and edited the content as needed and take full responsibility for the content of the published article.

## Financial disclosure statement

This work was supported by the Gus Schumacher Nutrition Incentive Program - Produce Prescription Program, project award no. 2022-70422-37754, from the U.S. Department of Agriculture's National Institute of Food and Agriculture.

## CRediT authorship contribution statement

**Alyssa Castilla:** Writing – review & editing, Writing – original draft, Formal analysis. **Tafere Belay:** Writing – review & editing, Formal analysis. **Deborah Gauck:** Writing – review & editing, Visualization, Project administration, Funding acquisition, Conceptualization. **Nicole Stendell-Hollis:** Writing – review & editing, Writing – original draft, Formal analysis, Data curation, Conceptualization.

## Declaration of competing interest

The authors declare the following financial interests/personal relationships which may be considered as potential competing interests: The authors confirm the work is original, not under review or published elsewhere, approved by all authors, and includes financial disclosures.

## Data Availability

The authors do not have permission to share data.
